# Short-Term Blood Pressure Variability among Young Adults at High or Low Risk for Depression

**DOI:** 10.3390/jcm13164640

**Published:** 2024-08-08

**Authors:** Balázs Bence Nyárády, Miklós Vértes, Edit Dósa, Xiao Yang, Charles J. George, Enikő Kiss, Ildikó Baji, Krisztina Kapornai, Maria Kovacs

**Affiliations:** 1 Heart and Vascular Center, Semmelweis University, 1122 Budapest, Hungary; 2 Medical Imaging Center, Semmelweis University, 1082 Budapest, Hungary; 3 Department of Psychology, Old Dominion University, Norfolk, VA 23529, USA; 4 University of Pittsburgh Medical Center, Pittsburgh, PA 15213, USA; 5 Department of Pediatrics and Pediatric Health Center, Child and Adolescent Psychiatry Unit, University of Szeged, 6725 Szeged, Hungary; 6 Faculty of Health Sciences, Semmelweis University, 1088 Budapest, Hungary; 7 Department of Psychiatry, University of Pittsburgh School of Medicine, Pittsburgh, PA 15213, USA; kovacs@pitt.edu

**Keywords:** blood pressure, short-term blood pressure variability, cardiovascular risk, childhood-onset depression, depressive episodes, hypertension prevention

## Abstract

**Background:** Depression has been shown to have adverse effects on blood pressure (BP) and is associated with high blood pressure variability (BPV). In turn, high short-term BPV has been related to eventual cardiovascular risk. But it is not clear how early in adulthood the detrimental effects of depression on BPV may be discerned, if being at high risk for depression also compromises BPV, and whether the clinical features of depression moderate its adverse effects. We investigated these three issues among young adults using an office-like setting. **Methods:** In total, 218 subjects with a history of childhood-onset major depressive episodes (probands), 206 never-depressed full biological siblings of the probands (high-risk siblings), and 166 emotionally healthy unrelated controls received a psychiatric evaluation and three standardized-sitting BP measurements 5 min apart. Short-term BPV was defined as the maximum difference between measures (range) for each case. The statistical methods included analyses of variance/covariance, chi-square tests, and multiple regression. **Results:** Systolic and diastolic BP decreased over consecutive measurements (*p* < 0.001). After controlling for age, the probands, siblings, and controls did not differ significantly in terms of BPV. However, the number of lifetime depressive episodes did predict the diastolic BP range (*p* = 0.005): probands with the highest number of depressive episodes had the largest short-term diastolic BPV. **Conclusions:** On a group level, the adverse effects on BPV of having experienced or being at high risk for depression are not yet evident during young adulthood. However, the number of major depressive episodes, which is an index of lifetime depression burden, predicts higher BPV. Thus, BPV monitoring for young adults with clinical depression histories could be part of an early intervention program to reduce the risk of eventual cardiovascular disease.

## 1. Introduction

Clinical depression has been recognized as an independent risk factor for cardiovascular disease [1]: it affects cardiovascular regulation [2], impairs autonomic functioning [3], and predicts hypertension [4], coronary heart disease, and all-cause mortality [5]. While the exact mechanism whereby depression affects cardiovascular health and targets organ damage is only partly understood [6], atypical blood pressure (BP) is believed to be one physiological link [4]. In turn, high blood pressure variability (BPV), one of the defining features of atypical BP, has also been shown to prognosticate cardiovascular problems, eventual multi-systemic damage, and even all-cause mortality [4,6,7,8,9]. Blood pressure variability can be determined within various time frames (ultra short-term, such as beat-to-beat; short-term, such as <24 h; long-term, such as visit-to-visit) and quantified via several metrics (e.g., standard deviation [SD], range, coefficient of variation, independent variation, mean true variability) [8,10,11].

As recent reviews reveal [8,10,11], a substantial amount of research has been conducted on BPV and its prognostic utility is widely accepted. There is also an emerging body of literature, that points to an association between BPV and depression. For example, Shahimi et al.’s [12] recent review addressed the relationship between BPV and mental disorders, including depression: they identified 12 studies that met their selection criteria, including six that examined patients with depression or depressive disorders. The review concluded that, in general, individuals with mental illness are significantly likely to have increased BPV regardless of age. Specifically, depressed individuals were found to have higher short-term BPV [12].

However, while the studies of depression and BPV have covered a wide age range, the typical sample generally comprises middle-aged or older individuals. Given that depression (as well as most other major mental disorders) initially emerges in adolescence or earlier [13], it is important to know whether its detrimental effects on BPV are evident already during young adulthood. Relatedly, little is known about whether the clinical features of a person’s depression (e.g., number of episodes) contribute to its detrimental effects. And while individuals with a family history of depression are at high risk of developing depression themselves [14], there is no information as to whether being at high risk (versus having already had depression) also predicts elevated BPV.

Finally, given that increased BPV predicts multiple adverse cardiovascular outcomes separately and independently of the average BP, and thus has considerable value [10,11], one question is why this index has not been embraced in everyday clinical practice [11]. One contributing factor may be that there is no standardized protocol for the measurement of BPV [10,12]; alternatively, the various approaches used in research settings may be too cumbersome or burdensome. Indeed, Schutte et al. [11] have noted that despite the dynamic nature of BP and advances in measurement techniques, the most important clinical decisions are usually based on three, static, office-based BP measures using the upper-arm-cuff method.

To study the effect of depression on BPV among young adults, we therefore designed a protocol that should be easy to reproduce in typical clinical settings. We focused on short-term (<24 h) BPV and measured BP in a standardized manner via the upper-arm-cuff method. We studied a sample of young adults who had psychiatrically diagnosed childhood-onset major depressive disorder (referred to as probands from here on), their full biological siblings who never had depression (a group at high risk for depression), and emotionally normative controls free of lifetime depression. Further information on the relationship between clinical depression and BPV may help to identify and address both mental health and cardiovascular issues as early as possible across the age span and thereby improve overall health outcomes later in life.

## 2. Patients and Methods

### 2.1. Subjects

Subjects for the present study were ascertained by contacting individuals who have participated in a prior study of juvenile-onset depression and made their contact information available to future research. The prior study recruited probands and their siblings in Hungary from 23 child mental health facilities, serving both urban and rural areas [15], from the year 2000 to 2006 for a genetic and clinical study. Probands had to meet the following criteria: have had a current or recent DSM-IV (Diagnostic and Statistical Manual of Mental Disorders, fourth edition) [16] major depressive or dysthymic episode; be 7–14 years of age; be free of intellectual disability and major medical disorders; and have at least one biological parent and a 7–17.9-year-old full biological sibling available for the study [17,18]. Controls were recruited from schools in the areas in which most of the probands resided. For more details on the recruitment of school-based controls, please see a previous publication [17].

To gather the overall sample for the present investigation, we re-contacted all available probands, siblings (age 18 or older), and controls. After explaining the study and receiving informed consent, we assessed all those who wanted to participate. We then enrolled all probands; that is, all the young adult subjects with a history of childhood-onset major depressive episodes (*n* = 218), the full biological siblings of the probands (high-risk siblings) who had no history of depressive disorders (*n* = 206), and the controls who have continued to remain free of major psychiatric disorders (*n* = 166).

Table 1 includes characteristics of the samples in the current study. As shown, probands were older than the siblings and controls; and siblings were older than the controls. There were more females among the probands and siblings than the controls (Table 1). The current research study was approved by the Hungarian National Ethical Committee as well as the institutional review boards of the University of Pittsburgh and the Hungarian clinical research sites. All subjects provided written informed consent.

### 2.2. Assessments

Subjects took part in a larger project that involved a psychiatric assessment and cardiovascular evaluation, including measurements of BP. Psychiatric diagnoses were derived according to DSM criteria [16,19]. The information needed was obtained in direct interviews with subjects by trained clinicians via the semi-structured Interview Schedule for Young Adults: Follow-Up Diagnostic version (ISYA-D), which is an age-appropriate modification of the tools used with this sample when they were pre-adults [18]. Operational criteria were used to date on- and offsets of psychiatric disorder episodes, which was necessary in order to determine episode numbers for any given disorder [20]. Final diagnoses, including confirmation of number of episodes, were based on consensus among senior diagnosticians. Subjects also completed the self-rated Beck Depression Inventory II (BDI-II) [21], which is a widely used, reliable, and valid index of the severity of current (past 2 weeks) depressive symptoms. In the current article, we report only on outcomes of psychiatric assessment and BP measurements.

### 2.3. Procedures

Subjects were asked to abstain from caffeine, alcohol, and tobacco for 1 h prior to BP measurements. The lab assistants followed a written protocol in assessing BP. After a brief rest period, three sitting brachial BP measurements were taken on each subject at 5 min intervals. Subjects were asked to sit on a chair with their arms resting at the level of the heart and both feet on the floor. All BP measurements were taken on the right arm by a trained assistant with an Omron M6 digital BP machine (Omron Corp., Kyoto, Japan). Short-term BPV was defined as the maximum difference between measures (range).

### 2.4. Statistical Analysis

Statistical analyses were conducted using SAS 9.4 software. One-way analyses of variance (ANOVA) and *χ*^2^ tests were used to compare continuous and categorical variables across groups. Data were screened for outliers, and ANOVA was used to examine group differences in average BP and short-term BPV. Then, we used analysis of covariance (ANCOVA) to examine group effects while sequentially controlling for variables known to influence BP, namely sex, age, body mass index (BMI), and smoking (yes/no). To account for dependent observations (probands and siblings were not independent), ANOVA and ANCOVA were estimated using linear mixed-effects models with random intercepts for each family. Least-squares mean estimates were used to perform pairwise comparisons of groups. A power analysis of a one-way ANOVA using the current sample sizes showed that, at 80% power, we could detect an overall significant *F*-test with the largest pairwise mean difference as little as 0.29 SD (i.e., a medium effect size). This effect size represents a difference of 1.7 mm Hg in systolic BPV and 1.6 mm Hg in diastolic BPV.

In the second set of analyses, confined to probands, BP range as the dependent variable was regressed on three separate variables that mirror clinical features of depression history: number of depressive episodes, age at onset of the first depressive episode, and percent of lifetime spent in depression, controlling for sex, age, BMI, and smoking. Effect sizes of predictors were estimated by partial *R*^2^ in the mixed-effects models and by partial *η*^2^ in the regression models.

## 3. Results

### 3.1. Characteristics of the Groups

As shown in Table 1, the three groups did not differ in BP medication use. However, probands and siblings had larger BMIs and were more likely to be smokers than the controls. Not surprisingly, the BDI scores were higher in probands than in siblings and controls. Additionally, probands and siblings had similarly higher diastolic BP (mean of three readings) than the controls (Table 1).

Based on the psychiatric evaluations, 9.2% (*n* = 20) of the probands were in a depressive episode at assessment and the rest were in remission; none of the siblings and controls were currently depressed (*χ*^2^ = 35.33, *p* < 0.001). Furthermore, while no controls were taking any psychotropic medication, 1.5% (*n* = 3) of the siblings and 4.1% (*n* = 9) of the probands were on psychotropic medication at the time of BP assessment (*χ*^2^ = 8.59, *p* = 0.014).

### 3.2. Blood Pressure Characteristics and Variability

As shown in Table 1, unadjusted group differences in systolic BP means or ranges were not statistically significant (*F* [2, 586] < 0.5, *p* > 0.60). Adjusting for age, sex, BMI, and family clusters did not change the means (*F* [2, 413] = 0.27, *p* = 0.77) or ranges (*F* [2, 440] = 0.70, *p* = 0.50). While there was a significant group difference in mean diastolic BP (*F* [2, 586] = 7.29, *p* < 0.001), this effect was no longer significant after covarying for age. Overall, the three groups did not differ significantly in diastolic BP ranges either in an unadjusted model (*F* [2, 586] = 0.14, *p* > 0.80) or after adjusting for age, sex, BMI, smoking, and family clusters (*F* [2, 587] = 0.62, *p* = 0.54).

**Figure 1:**
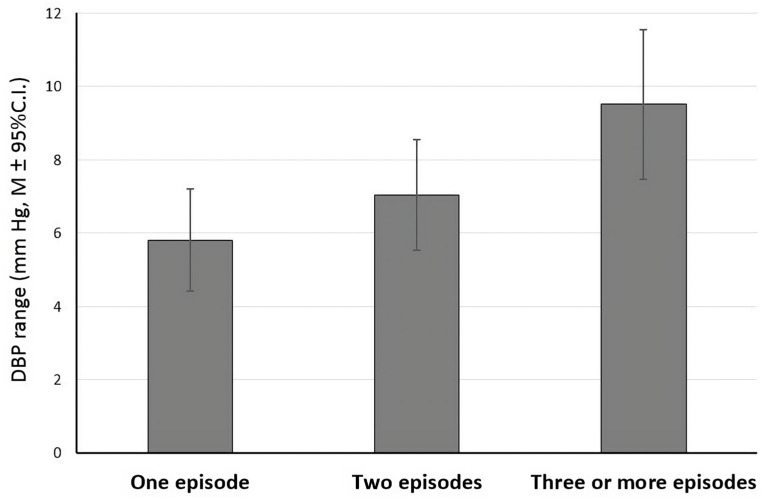
Number of lifetime depressive episodes and diastolic blood pressure range among probands (adjusted for sex, age, body mass index, and smoking). C.I.—confidence interval; DBP—diastolic blood pressure; M—mean.

The second set of analyses confined to probands revealed that the number of depressive episodes predicted the diastolic BP range, even after adjustment for covariates (*β* = 1.76, *t* [210] = 2.87, *p* = 0.005, *ηp*^2^ = 0.039) (Figure 1). Namely, probands with the highest number of depressive episodes had the largest diastolic BPV. For example, for probands with one depressive episode, the diastolic BP range was 5.86 (SD = 4.8), while for probands with three or more depressive episodes, the diastolic BP range almost doubled at 9.53 (SD = 12.0). The importance of the number of depressive episodes received partial support when we modeled systolic BPV: while the overall model was not significant (*F* [5, 210] = 1.13, *p* = 0.34), there was a trend for the number of depressive episodes to be related to larger systolic BP ranges (*β* = 0.98, *t* [210] = 1.82, *p* = 0.071).

Additional analyses showed that age at onset of the first depressive episode did not predict the systolic BP range (*β* = −0.01, *t* [210] = −0.02, *p* = 0.99) or the diastolic BP range (*β* = 0.11, *t* [210] = 0.44, *p* = 0.66). Similarly, percentage of lifetime spent in depression had no significant effects on the systolic BP range (*β* = 0.04, *t* [210] = 1.21, *p* = 0.23) or the diastolic BP range (*β* = −0.02, *t* [210] = −0.37, *p* = 0.71). Finally, psychiatric variables appear to have had minimal effects on the BP parameters that were examined. Specifically, subjects who were taking psychotropic medication and those who were not on medication did not differ significantly in either systolic or diastolic BP ranges (*F* < 1.77, *p* > 0.19).

## 4. Discussion

In the present study, we investigated whether the harmful impact of depression on short-term BPV, which has been reported in mostly middle-aged and older cohorts [12], can also be detected among young adults in their twenties. To extend the study of depression and BPV, we also examined young adults at familial risk for depression and whether the clinical features of depression played a role in BPV. The characteristics of our probands are similar to those previously reported for depressed patients, including higher rates of smoking, lower levels of physical activity, and higher BMI than controls [22,23]. Our finding of declining BP with consecutive measurements (the “white-coat effect”) in all groups is also in line with the literature [24,25].

A motivator for the present study was the review by Shahimi et al. [12], which concluded that depression is associated with increased BPV (regardless of age). However, we failed to support that conclusion. We found that young adults with diagnosed depression histories, never-depressed individuals at high familial risk for depression (the siblings), and controls did not differ in either systolic or diastolic BPV. Thus, pathological BPV as a function of depression is not yet detectable when individuals are in their twenties, possibly because that outcome requires a certain level (or amount) of lifetime depression burden that can be reached only with more advanced age. However, because most of the probands were in remission from their last episode of depression, an alternative explanation for our finding is that current rather than past depression (depression history) is the decisive factor in pathological short-term BPV. Post hoc analyses provide some support for the latter explanation: differing in the expected direction, although not significantly so, probands who were experiencing depression (*n* = 20) compared to those in remission (*n* = 197) had both higher diastolic BPV (*M* = 9.0, SD = 15.3 and *M* = 6.8, SD = 5.5, respectively) and higher systolic BPV (*M* = 12.2, SD = 11.5 and *M* = 8.3, SD = 5.1, respectively). However, the *n* = 20 subset did not provide sufficient power to detect across-group differences in BPV.

The duration and recurrence of depressive episodes may also contribute to the cardiovascular effects of depression [26,27]. Relatedly, we found that short-term BPV was predicted by how many times a person had a diagnosable depression (number of depressive episodes) but not by how much that person’s life had been taken up by depression (percent of one’s lifetime spent in depression). Thus, BPV appears to be particularly vulnerable to disruptions or discontinuities in functioning, which are mirrored by the starts and ends of discrete episodes of depression, whereas the extent of exposure to depression had only a scant discernable effect. However, as noted above, being a young adult constrained the extent of potential exposure to depression. On the other hand, the relationship between number of depressive episodes and BPV may also derive from the behavioral concomitants of depression, including higher rates of smoking and lower levels of physical activity, both of which are known to affect BP parameters [22,23].

Another feature of depression, age at first onset, had no discernible effect on BP parameters. This result may reflect that our probands had their depression onset in childhood, which yielded a restricted age range. By studying a broader age group and following samples to older ages, at which time the effect of depression on cardiovascular risk becomes more evident, future research will be in a better position to address how the various clinical features of depression contribute to atypical BPV. Early identification of and intervention with depression-prone cohorts may forestall atypical BPV and thus perhaps reduce eventual cardiovascular problems.

Finally, we note that the association of BPV and depressive episodes in our study was evident only for diastolic BPV. Sible et al. [28] likewise found that depression symptoms and diastolic (but not systolic) BPV were related and noted that diastolic BPV is believed to reflect factors such as endothelial dysfunction and sympathetic autonomic nervous system (ANS) over-reactivity. Indeed, depression is known to be associated with atypical ANS functioning, as reflected by an overall reduction in parasympathetically mediated cardiac vagal control [29]. Relatedly, there is evidence that short-term BPV increases are primarily under sympathetic control [30]. Alternatively, given the evidence that probands have adverse levels of metabolic syndrome components (e.g., lower high-density lipoprotein, higher triglycerides) [31], metabolic syndrome could have mediated the relationship between BPV and depressive episodes.

Our study has several strong features, including a large clinical sample, a sample of high-risk siblings, and standardized psychiatric evaluations by trained clinicians. Additionally, we selected BP range as our measure of BPV because it is clinically meaningful and understandable to healthcare professionals; this measure of variability has also been used in other recent studies (e.g., Sible et al. [28]). Although researchers often prefer more complex metrics of short-term BPV than the range, the alternative indices tend to be highly inter-correlated, as reported by Schutte et al. [11]. In our own dataset, for example, the SD of the mean (one index of variability) correlated with both systolic and diastolic BP range at *r* = 0.99 (*p* < 0.01). Our monitoring method of three consecutive measurements at 5 min intervals can be performed quickly and effectively in an ambulatory office setting and serve as an adjunct to home-based assessment. However, in spite of our study’s strengths, the results should be considered in light of the limitation that BP was only sampled on a single day. Assessments spread over several days may provide a more accurate picture of BPV and eventual cardiovascular risks. Another limitation is that the lab assistants may have differed in how precisely they followed the BP measurement protocol. This source of potential variability may be remedied in future studies by monitoring lab assistants’ behavior. It is worth noting that our study, like all cross-sectional studies, can uncover associations among the variables of interest, but cannot speak to causal relationships among them. Furthermore, whereas our study included one of the largest samples of young adults with childhood-onset depression, much larger samples are needed to detect very small effect sizes.

In conclusion, the disruptive effect of depression on BP is not yet discernible in young adults in their twenties. However, a greater lifetime burden (indexed by episode number) predicts higher BPV. Thus, BP monitoring for young adults with depression histories may help to identify those at elevated risk for eventual cardiovascular problems and allow the implementation of preventive services.

## Figures and Tables

**Table 1:** Demographic, clinical, and blood pressure characteristics of the samples.

Parameters	Probands (*n* = 218)	Siblings (*n* = 206)	Controls (*n* = 166)	*F* or *χ*^2^
Female, n (%)	103 (47.2) ^a^	108 (52.4) ^a^	62 (37.3) ^b^	8.54 *
Age at assessment (years), mean (SD)	25.1 (2.5) ^a^	24.3 (3.7) ^b^	21.7 (1.5) ^c^	73.61 ***
BMI (kg/m^2^), mean (SD)	24.65 (5.36) ^a^	24.83 (5.61) ^a^	23.16 (3.49) ^b^	6.02 **
Current smokers, n (%)	116 (53.5) ^a^	87 (42.4) ^b^	41 (24.7) ^c^	31.15 ***
Current BP medication, n (%)	2 (0.9)	3 (1.5)	2 (1.2)	0.26
Systolic BP (mm Hg)				
Average (SD)	112.2 (12.1)	111.8 (10.7)	111.4 (11.5)	0.24
Range (SD)	8.6 (6.0)	9.0 (5.6)	9.2 (6.1)	0.41
Diastolic BP (mm Hg)				
Average (SD)	73.0 (8.2) ^a^	73.4 (8.1) ^a^	70.4 (7.8) ^b^	7.29 **
Range (SD)	7.0 (7.0)	6.9 (4.4)	7.2 (5.4)	0.14
BDI-II score, mean (SD)	7.08 (8.15) ^a^	4.66 (5.61) ^b^	3.56 (4.22) ^b^	15.75 ***
Age at onset of first depressive episode (years), mean (SD)	10.4 (2.4)	n.a.	n.a.	n.a.
Number of depressive episodes, n (%)				
1	94 (43.1)	n.a.	n.a.	n.a.
2	80 (36.7)	n.a.	n.a.	n.a.
3 or more	44 (20.2)	n.a.	n.a.	n.a.
Percent of lifetime spent in depressive episodes, mean (SD)	12.24 (11.99)	n.a.	n.a.	n.a.

BDI-II—Beck Depression Inventory II; BMI—body mass index; BP—blood pressure; SD—standard deviation. Average and range of BP were calculated as the mean and the biggest difference among the three assessments in the sitting condition, respectively. All statistics are unadjusted. *, *p* < 0.05; **, *p* < 0.01; ***, *p* < 0.001. Superscript letters denote significant pairwise contrast at *p* < 0.05.

## Data Availability

The data presented in this study are available on request from the corresponding author. The data are not publicly available due to reasons pertaining to patient privacy.
